# Using Pre-existing Microarray Datasets to Increase Experimental Power: Application to Insulin Resistance

**DOI:** 10.1371/journal.pcbi.1000718

**Published:** 2010-03-26

**Authors:** Bernie J. Daigle, Alicia Deng, Tracey McLaughlin, Samuel W. Cushman, Margaret C. Cam, Gerald Reaven, Philip S. Tsao, Russ B. Altman

**Affiliations:** 1 Department of Genetics, Stanford University School of Medicine, Stanford, California, United States of America; 2 Division of Cardiovascular Medicine, Stanford University School of Medicine, Stanford, California, United States of America; 3 Division of Endocrinology, Stanford University School of Medicine, Stanford, California, United States of America; 4 National Institute of Diabetes and Digestive and Kidney Diseases, National Institutes of Health, Bethesda, Maryland, United States of America; 5 Department of Bioengineering, Stanford University School of Medicine, Stanford, California, United States of America; University of Illinois at Urbana-Champaign, United States of America

## Abstract

Although they have become a widely used experimental technique for identifying differentially expressed (DE) genes, DNA microarrays are notorious for generating noisy data. A common strategy for mitigating the effects of noise is to perform many experimental replicates. This approach is often costly and sometimes impossible given limited resources; thus, analytical methods are needed which increase accuracy at no additional cost. One inexpensive source of microarray replicates comes from prior work: to date, data from hundreds of thousands of microarray experiments are in the public domain. Although these data assay a wide range of conditions, they cannot be used directly to inform any particular experiment and are thus ignored by most DE gene methods. We present the SVD Augmented Gene expression Analysis Tool (SAGAT), a mathematically principled, data-driven approach for identifying DE genes. SAGAT increases the power of a microarray experiment by using observed coexpression relationships from publicly available microarray datasets to reduce uncertainty in individual genes' expression measurements. We tested the method on three well-replicated human microarray datasets and demonstrate that use of SAGAT increased effective sample sizes by as many as 2.72 arrays. We applied SAGAT to unpublished data from a microarray study investigating transcriptional responses to insulin resistance, resulting in a 50% increase in the number of significant genes detected. We evaluated 11 (58%) of these genes experimentally using qPCR, confirming the directions of expression change for all 11 and statistical significance for three. Use of SAGAT revealed coherent biological changes in three pathways: inflammation, differentiation, and fatty acid synthesis, furthering our molecular understanding of a type 2 diabetes risk factor. We envision SAGAT as a means to maximize the potential for biological discovery from subtle transcriptional responses, and we provide it as a freely available software package that is immediately applicable to any human microarray study.

## Author Summary

Though the use of microarrays to identify differentially expressed (DE) genes has become commonplace, it is still not a trivial task. Microarray data are notorious for being noisy, and current DE gene methods do not fully utilize pre-existing biological knowledge to help control this noise. One such source of knowledge is the vast number of publicly available microarray datasets. To leverage this information, we have developed the SVD Augmented Gene expression Analysis Tool (SAGAT) for identifying DE genes. SAGAT extracts transcriptional modules from publicly available microarray data and integrates this information with a dataset of interest. We explore SAGAT's ability to improve DE gene identification on simulated data, and we validate the method on three highly replicated biological datasets. Finally, we demonstrate SAGAT's effectiveness on a novel human dataset investigating the transcriptional response to insulin resistance. Use of SAGAT leads to an increased number of insulin resistant candidate genes, and we validate a subset of these with qPCR. We provide SAGAT as an open source R package that is applicable to any human microarray study.

## Introduction

Since their inception over 13 years ago [1], DNA microarrays have become a staple experimental tool used primarily for exploring the effects of biological interventions on gene expression. Microarrays have enabled a range of experimental queries, including a survey of gene expression across dozens of mammalian tissues [2], a comparison of human cancers in over 2000 tumor samples [3], and the identification of differentially expressed (DE) genes between pairs of conditions. Identifying DE genes is especially common, as it is often the first means of characterizing differences between two poorly understood conditions. As of 2009, there are publicly available microarray data for 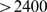 human conditions (at the Gene Expression Omnibus [4]). These data make possible a huge number of pairwise comparisons for DE gene analysis. Given this sizable opportunity for biological discovery, we focus our attention on the task of DE gene identification.

Microarrays are notorious for generating noisy or irreproducible data [5]–[8]. This is partially due to the inherent technical noise of the experiment, which can be modeled and often removed from the resulting data. However, biological noise also plays a significant role, and effects of this noise source are not as easily corrected [9]. A common solution to biological noise involves replicating the experiment many times in order to “average out” noise effects. In the context of DE gene prediction, we define a replicate as a biologically independent comparison of RNA levels between the experimental conditions of interest. Unfortunately, assay cost and a limited supply of biological material often limit the efficacy of a replication-based strategy. To circumvent these difficulties, we need analytical methods which increase DE gene prediction accuracy at no additional cost.

One inexpensive source of microarray replicates comes from prior experiments. In the last decade, researchers have generated data from hundreds of thousands of microarrays, and many of these are publicly available at repositories like the Gene Expression Omnibus (GEO). It is unlikely that any of these arrays (hereafter referred to as “knowledge”) represent exact replicates of data from a novel study (referred to as “data”), but a subset of these experiments may describe similar underlying biology and could be considered “partial replicates”. Because it is not clear *a priori* which of the prior experiments (if any) would qualify as partial replicates, pre-existing microarray knowledge cannot be used directly to identify DE genes in a novel dataset.

It is therefore worth considering indirect methods for using this knowledge. Two previously existing methods use microarray knowledge to compute more accurate variance estimates for each gene [10],[11]. Both methods replace sample variance estimates for each gene by gene-specific variances calculated across a compendium of microarrays from GEO. This approach was shown to be most useful with small data sample sizes, and no further benefits were seen when the microarray knowledge exceeded 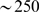 arrays. A different approach might involve identifying transcriptional modules: groups of genes that exhibit coordinated or correlated expression changes across a range of conditions. A complete and accurate understanding of module structure would reveal expression dependencies between genes, such that on average, genes in the same module would be coexpressed more often than genes chosen at random. Thus, knowledge of one gene's expression would confer information about the expression of the other genes in the module. Several studies [12]–[18] have used microarray knowledge to identify transcriptional modules. Of these, five have been tested on yeast datasets of 1000 arrays or fewer [12]–[14],[16],[17] and one has been applied to 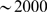 human cancer datasets [15]. Only one [18] was applied to a diverse human microarray knowledge set, in this case containing 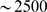 arrays. Given that tens of thousands of arrays are publicly available for some individual microarray platforms, a larger-scale identification of transcriptional modules is certainly possible.

Knowledge of transcriptional modules and their constituent genes is not directly applicable to DE gene identification, and most existing methods ignore these relationships. Of the few that provide a means to incorporate expression modules [19]–[21], none provide a mechanism for extracting these modules from large-scale microarray knowledge sets. Consequently, there is a need for a method that can identify relevant transcriptional modules from huge compendia of microarray knowledge and use this information to better predict DE genes.

In this work, we present the SVD Augmented Gene expression Analysis Tool (SAGAT), a mathematical approach that identifies expression modules from microarray knowledge and combines these with novel data to identify DE genes. To accomplish these tasks, SAGAT employs Singular Value Decomposition (SVD) in concert with pseudoinverse projection. SVD has been used previously to decompose microarray knowledge into mathematically independent transcriptional modules (eigengenes) and the corresponding independent cellular states where these modules are active (eigenarrays) [22]. Most non-SVD module-finding methods identify discrete modules where module membership for each gene is a binary feature. In contrast, SVD assigns a continuously-valued weight for each gene, which allows varying strengths of coexpression to be present in the same module and genes to be part of multiple modules. SVD models the expression of each gene as a linear combination of the eigengenes' expressions, and a number of studies have used this technique to define modules on smaller scales. Raychaudhuri et al. [23] and Alter et al. [22] each initially applied SVD (the former in the form of PCA) to yeast time course data to identify fundamental modes of expression response that vary over time. The latter study also demonstrated the ability of SVD to remove noise or experimental artifacts present in the data. Shortly thereafter, Troyanskaya et al. [24] used SVD to identify eigengenes in gene expression data for the purposes of missing value estimation. Alter and colleagues subsequently employed generalized [25] and higher order [26] versions of SVD for the integration and decomposition of heterogeneous microarray datasets. Horvath and Dong [27] used SVD of microarray data in combination with coexpression analysis to generate eigengene coexpression networks. Finally, in a large scale study, SVD was shown to reduce noise when used in the integration of disparate microarray datasets [28].

The technique of pseudoinverse projection has also previously been applied to genome-scale data. Alter and Golub demonstrated the utility of SVD coupled with pseudoinverse projection by reconstructing one genomic dataset in terms of the eigenarrays of another [29]. This enabled the observation of a set of cellular states in one dataset that were also manifested in the other. Subsequent work used pseudoinverse projection in concert with an alternative matrix decomposition technique (non-negative matrix factorization) to classify gene expression states of one organism in terms of another [30]. In the current work, using SAGAT, we combine SVD-derived modules, pseudoinverse projection, and a rigorous statistical model to adjust gene expression error estimates in a dataset of interest. This yields a knowledge-informed differential expression score for each gene.

We demonstrate SAGAT in several ways. First, we investigate whether transcriptional modules are readily detectable in a large compendium of microarray knowledge. Second, we test SAGAT on a range of simulated datasets to assay its performance with respect to a known gold standard. Third, we evaluate SAGAT's ability to increase DE gene predictive power in three highly replicated real world datasets. Finally, we apply SAGAT to a new human dataset investigating transcriptional profiles in the setting of insulin resistance (IR), a risk factor for type 2 diabetes. Though a known relationship exists between obesity and insulin resistance [31],[32], it is not always consistent [33],[34]; in addition, many studies characterizing IR do not deconvolve the effects of obesity [35]. This novel microarray dataset builds upon previous work [35]–[37] to investigate obesity-independent transcriptional effects of insulin resistance. We illustrate the improved sensitivity of SAGAT over existing methods by identifying IR candidate DE genes, and we validate a subset of these using quantitative PCR assays. Results of this analysis contribute to a more comprehensive molecular understanding of human insulin resistance.

## Results

### Modularity of Gene Expression Data

To demonstrate that transcriptional modules are detectable in a multi-condition microarray knowledge compendium, we characterized the degree of modularity in a collection of 4440 arrays from the HGU95Av2 platform. We consider an expression module a group of genes exhibiting coordinated expression across some subset of the entire compendium. Genes in such a group will have relatively large positive or negative pairwise covariances; thus, degree of modularity refers to the number of genes in the compendium that belong to one or more groups of significantly covarying genes.

**Figure 1:**
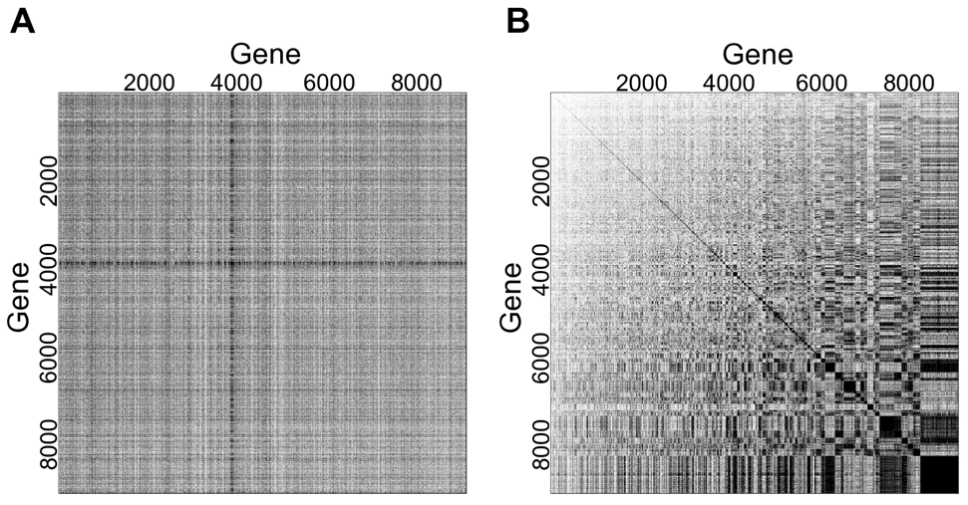
Modularity characterization of HGU95Av2 compendium. (A) All pairwise covariances were calculated between 9105 genes; entries whose absolute values were greater than .25 were set to one and colored black (set to zero and colored white otherwise). (B) Binarized covariance matrix after hierarchical biclustering (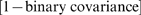 distance metric and complete linkage) to identify coordinated expression modules. The observed modularity is not due to chance, as an identical procedure applied to a randomized expression matrix showed no covariance blocks larger than a few genes (not shown).

Figure 1A displays a binarized representation of the sample covariance matrix for the entire HGU95Av2 compendium, whereby each covariance value whose magnitude is 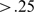 is colored black (white otherwise). This matrix was then subjected to hierarchical biclustering (Figure 1B), which resulted in many blocks of nonzero binary covariance, ranging in size from a few genes to nearly 1000. Furthermore, this covariance pattern does not appear to be due to chance, as the biclustering results from 100 randomized knowledge matrices (see Materials and Methods) showed no covariance blocks exceeding a 15 gene cutoff.

To parameterize a simulation study (details below), we used a 1000-gene compendium to characterizee the mean number of genes per module, the mean percentage of DE genes found in modules, and the mean percentage of non-DE genes found in modules. This was achieved by subsetting the HGU95Av2 compendium and coupling it with a human prostate cancer dataset [38]. The mean number of genes per module was 15, the mean percentage of DE genes found in modules was 60% (673/1122), and the mean percentage of non-DE genes found in modules was 47% (3752/7983). These values were employed in the simulation study that follows.

### Singular Value Decomposition (SVD) of Gene Expression Data

SVD identifies eigengenes whose expression is mutually orthogonal across all arrays in the compendium. To demonstrate that mathematical orthogonality correlates with biological orthogonality (as manifested by biologically independent eigengenes), we performed a Gene Ontology (GO) term enrichment analysis of a subset of the eigengenes from the HGU95Av2 compendium (using the gene weights of each eigengene as scores). Table 1 displays the top three significant Biological Process terms with fewer than 500 annotated genes for eigengenes 1–5, 10, 20, 50, and 200. The terms within each eigengene are largely consistent, and each eigengene describes a relatively distinct biological process. We note that there is not an absolute correspondence between the modules displayed in Figure 1B and the eigengenes identified by SVD, as the methods used to identify these structures are algorithmically different. However, we detected substantial overlap in the enriched Biological Process terms associated with the largest covariance modules and highest ranking eigengenes (e.g. the largest module and first eigengene were both strongly enriched for translation and biosynthesis terms).

**Table 1:** GO Biological Process enrichment of HGU95Av2 compendium eigengenes.

Eigengene	Term ID	Name	# genes	p-value
1	GO:0006412	Translation	271	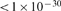
	GO:0009059	Macromolecule biosynthetic process	431	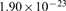
	GO:0006396	RNA processing	269	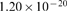
2	GO:0000278	Mitotic cell cycle	203	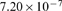
	GO:0022403	Cell cycle phase	223	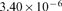
	GO:0000279	M phase	172	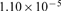
3	GO:0007155	Cell adhesion	494	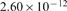
	GO:0006955	Immune response	481	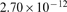
	GO:0000902	Cell morphogenesis	305	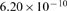
4	GO:0042110	T cell activation	86	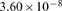
	GO:0046649	Lymphocyte activation	128	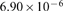
	GO:0045321	Leukocyte activation	145	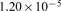
5	GO:0022403	Cell cycle phase	223	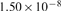
	GO:0000278	Mitotic cell cycle	203	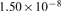
	GO:0007067	Mitosis	131	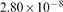
10	GO:0006941	Striated muscle contraction	27	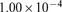
	GO:0016567	Protein ubiquitination	43	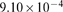
	GO:0006936	Muscle contraction	130	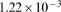
20	GO:0006323	DNA packaging	177	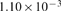
	GO:0006997	Nuclear organization and biogenesis	22	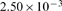
	GO:0006325	Establishment of chromatin architecture	171	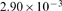
50	GO:0008354	Germ cell migration	6	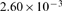
	GO:0050764	Regulation of phagocytosis	6	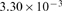
	GO:0007067	Positive regulation of phagocytosis	6	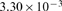
200	GO:0009187	Cyclic nucleotide metabolic process	35	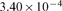
	GO:0009605	Response to external stimulus	424	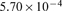
	GO:0030193	Regulation of blood coagulation	11	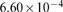

A Kolmogorov-Smirnov (K-S) test was used to evaluate the significance of enrichment. The member genes of each eigengene were ranked by the absolute value of their weights and the K-S test was run on all Biological Process terms. The top three terms with a p-value of .05 or smaller (and fewer than 500 annotated genes) are displayed.

### Simulation Study

**Figure 2:**
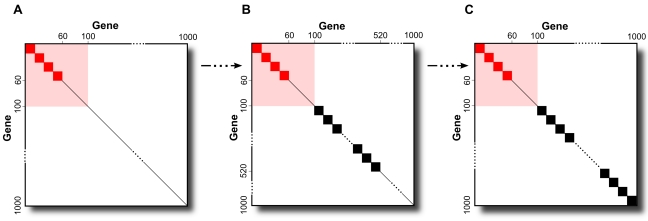
Modularity structures of simulated microarray compendia. Simulated compendia ranged from containing only DE gene modules (A) to having DE gene plus the maximum number of non-DE gene modules (C). (B) This configuration represents a conservative approximation of the structure of a real biological dataset (see Discussion).

We first tested the validity of the SAGAT model using simulated data. We simulated knowledge compendia with structures ranging from that shown in Figure 2A, where 60 of the 100 DE genes are in 15-gene modules and none of the 900 non-DE genes are, to that shown in Figure 2C, where the same number of DE genes are in modules and all 900 non-DE genes are also. Figure 2B depicts a modularity structure that is approximately equivalent to that of the prostate cancer dataset, where 60% of prostate cancer DE genes are found in modules and 47% of non-DE prostate cancer genes are found in modules.

After running SVD on each simulated compendium to calculate the appropriate **W** matrix, we tested SAGAT on all combinations of data and knowledge. As SAGAT relies on a single parameter specifying the number of eigengenes (*M*), we first estimated the optimal value for this parameter by trying all possible values on several configurations of data and knowledge (results not shown). The best performance was achieved with 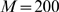; we used this value for all subsequent simulation runs.

**Figure 3:**
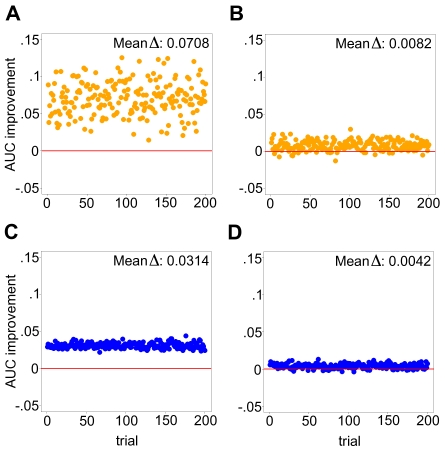
SAGAT performance on four simulated data-knowledge configurations. In each panel, both SAGAT and the fold change metric were applied to 200 simulated datasets consisting of either one [(A) and (B)] or 15 replicates [(C) and (D)]; the AUC improvement achieved by SAGAT over fold change is displayed for each. In (A) and (C), a simulated knowledge compendium matching Figure 2A was used by SAGAT; in (B) and (D) the simulated knowledge corresponds to Figure 2B. On average, SAGAT outperforms fold change in all four cases with the mean improvement located at the top of each panel. The simulated knowledge structure for (B) and (D) represents a conservative approximation of the structure of a real biological dataset (see Discussion).

Figure 3 displays results from running SAGAT on two compendia with modularity structures identical to Figures 2A (3A,3B) and 2B (3C,3D) coupled with datasets having either one or 15 replicates. The mean AUC improvement over the fold change metric (Mean 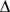), ranging from .0042 to .0708, is shown. Within the range of structures bounded by these two compendia and for both sample sizes, SAGAT consistently improves the AUC of DE gene prediction. The two trends observed are: (1) increasing performance improvement with decreasing numbers of array replicates, and (2) increasing performance improvement with decreasing numbers of non-DE gene modules. Performance begins to degrade below that of fold change if the simulated compendia adopt modularity structures between those of Figures 2B and C (results not shown), but we have evidence suggesting that the modularity of real world datasets resemble configurations falling between Figures 2A and B (see Discussion).

To demonstrate that use of SAGAT could yield improved statistical power without concurrently increasing the false positive rate of prediction, we repeated the above experiments using true positive rate (TPR) evaluated at a fixed false positive rate (FPR) of .05 (in place of AUC). These results are shown in Figure S3, and the performance improvements with respect to fold change closely resemble those displayed in Figure 3.

### Highly Replicated Real Datasets

To evaluate SAGAT performance on real data, we tested it on subsets of three highly replicated human microarray datasets (see Materials and Methods for details). As a gold standard, we used either the fold change or *limma* t [39] metrics to identify significant DE genes from each dataset in its entirety; this resulted in 1122 (12.3%), 588 (4.4%), and 6002 (29.9%) DE genes for the prostate cancer, letrozole treatment (GEO ID: GSE5462), and colorectal cancer (GSE8671) datasets, respectively.

**Figure 4:**
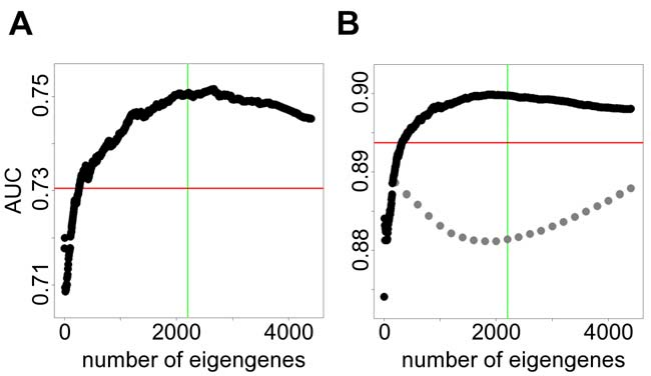
Identifying the optimal number of eigengenes. (A) and (B) show SAGAT performance versus varying numbers of eigengenes (parameter *M*) when applied to two non-overlapping four-array subsets of the prostate cancer dataset. The HGU95Av2 knowledge compendium was used for this task. The red horizontal lines denote the performance of the fold change metric. In both cases, *M* set to approximately half the number of arrays in the compendium leads to the best performance; this point is marked with green vertical lines. In (B), the light gray points display SAGAT performance when using a randomized knowledge compendium; this suggests that SAGAT improvement over fold change is not due to chance.

After downloading the three corresponding knowledge compendia (minus the highly replicated datasets) and running SVD on each, we determined the optimal number of eigengenes by training on the prostate cancer dataset. Figure 4 shows results of SAGAT run on two non-overlapping subsets of this dataset and the HGU95Av2 compendium while varying the number of eigengenes (parameter *M*). The AUCs of the fold change metric are displayed as red horizontal lines. SAGAT outperforms fold change for many values of *M*, and for both subsets there is a distinct maximum in the AUC curve for a particular value of the parameter. For these two subsets and several others tested (not shown), the optimal value for *M* is approximately half the number of arrays in the compendium. We used this value for subsequent analyses on all datasets and compendia, which translates to 2220, 7238, and 6108 eigengenes for the HGU95Av2, HGU133A, and HGU133plus2.0 platforms, respectively. To show that SAGAT's performance as a function of *M* was not due to chance, we randomized the expression values of the compendium and re-ran the same test in Figure 4B. These results are shown in gray. In this case, SAGAT never outperforms fold change, suggesting that the performance improvement from the original compendium is not spurious.

**Figure 5:**
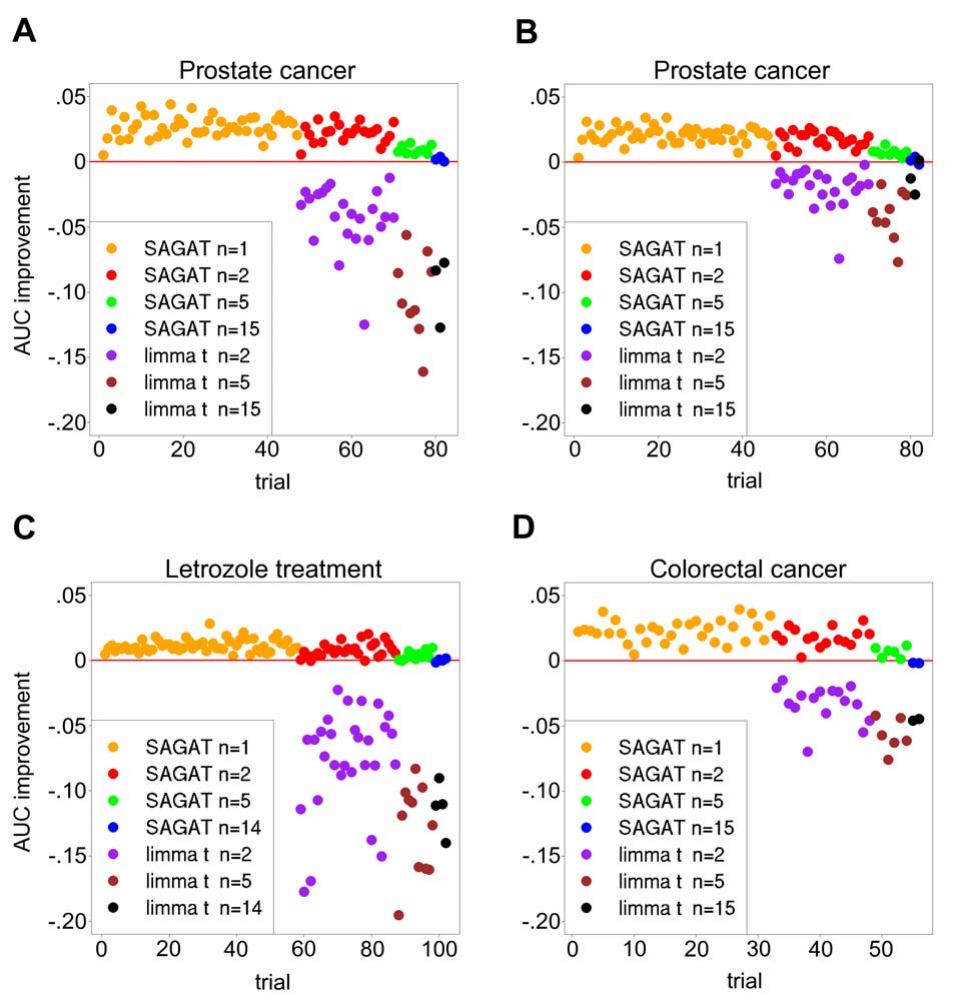
SAGAT, fold change, and limma *t* performance on subsets of three highly replicated human datasets. In each panel, the gold standard was defined as the top 1122, 588, and 6002 highest scoring genes for the prostate cancer, letrozole treatment, and colorectal cancer datasets, respectively. (A) The fold change metric was used to rank genes for the gold standard. SAGAT (with HGU95Av2 compendium), fold change, and limma *t* were run on all combinations of n = 1, 2, 5, and 15 replicate subsets and the performance improvement of SAGAT and limma *t* over fold change displayed. Limma *t* requires two or more replicates to score genes. (B) Identical conditions as (A), with the limma *t* metric used to rank gold standard genes. (C) and (D) Similar plots for the letrozole treatment (HGU133A compendium) and colorectal cancer (HGU133plus2.0 compendium) datasets, respectively. Fold change was used to rank gold standard genes in these panels. In all cases, SAGAT performs as well or better than the fold change and limma *t* metrics, while the limma *t* nearly always yields the poorest performance.

Next we applied SAGAT to multiple subsets of each of the three datasets. Figure 5 displays the performance of SAGAT coupled with the appropriate **W** matrices. For comparison, we feature AUC differences with respect to fold change of both SAGAT and the *limma* t-statistic. Figures 5A and B display performance on the prostate cancer dataset using a fold change and *limma* t-derived gold standard, yielding mean AUC improvements of .023 and .018, respectively. Given that the relative performance trends are similar, Figures 5C and D show performance on the letrozole treatment and colorectal cancer datasets using only the fold change-derived gold standard, yielding AUC improvements of .009 and .019, respectively. In all three datasets, irrespective of sample size, SAGAT nearly always improves the AUC over fold change; in cases where this does not occur, AUC is left essentially unchanged. In contrast, the t-statistic consistently lowers the AUC of DE gene prediction and is not applicable when the number of replicates is 1. Though the *limma* t performance improves when using a *limma* t gold standard, it is still unable to outperform the other two metrics. AUC improvement for SAGAT generally decreases with increasing sample size, and the improvement is largest for the prostate cancer and colorectal cancer datasets.

To express the performance of SAGAT in a more tangible form, we estimated the effective number of arrays added by using the method. Table 2 shows results for each of the three highly replicated datasets at four initial sample sizes. On average, with one exception in 12 tests, use of SAGAT always increased the effective number of arrays. In some cases, this improvement was quite significant: a two-array prostate cancer subset coupled with SAGAT effectively performed as well as a 4.72 array dataset. As before, the number of arrays added generally decreases with increasing sample size.

**Table 2:** Effective number of arrays when using SAGAT on three human datasets.

	Prostate cancer			Letrozole treatment*			Colorectal cancer		
# Initial arrays	Min	Mean	Max	Min	Mean	Max	Min	Mean	Max
2	2.32	3.66	4.72	2.28	2.90	4.24	2.20	2.92	3.59
4	4.43	5.66	6.59	3.97	4.74	5.81	4.13	4.92	5.56
10	10.77	11.21	11.91	9.95	10.52	11.27	10.13	10.68	11.22
30	30.14	30.87	31.70	29.45	30.08	30.58	28.89	29.00	29.10

AUC values from Figure 5 were converted to effective numbers of arrays by creating a “standard curve” of AUC versus sample size and interpolating (see Materials and Methods section for details).

*Results in the last row of the Letrozole treatment section were calculated using 28 initial arrays.

As with the simulated data, we also repeated the highly replicated dataset experiments using TPR calculated at an FPR of .05 as an evaluation metric. These results are displayed in Figure S4, and the performance improvements very closely resemble those shown in Figure 5.

### Comparison to Related Method

We evaluated the GEO method (both standard and “voting” methods) on the prostate cancer dataset and HGU95Av2 compendium and compared its performance to SAGAT. Figure S1 shows the results, which demonstrate that SAGAT (and fold change) outperform the GEO method in much the same way as when compared to the *limma* t-statistic above.

We also measured the sensitivity of SAGAT performance to compendium size. As Figure S2 shows, SAGAT continues to improve performance as the compendium increases to its full size. The performance begins to level off near 4400 arrays, but further improvement would still be expected with an even larger compendium.

### Insulin Resistance Dataset

Given encouraging performance of SAGAT on simulated and real human datasets, we applied it to an unpublished experimental dataset investigating expression differences between human insulin resistant and insulin sensitive adipose tissue. The obesity-independent relationship between insulin resistance and adipose gene expression has previously been characterized on a small scale [40], but no large-scale studies have attempted to decouple the effects of obesity from insulin resistance [35]. In this experimental design, patients were otherwise healthy and matched for levels of obesity; thus, we expected to identify more subtle expression changes associated with insulin sensitivity status.

As detailed in Materials and Methods, the same 12 pairs of RNA samples were applied to three different microarray platforms: Affymetrix, Agilent, and Illumina. We initially attempted to identify DE genes using the *limma* t metric on data from each platform individually. After correcting the results for multiple tests, we did not detect any significant genes at a .05 FDR cutoff. Next, we integrated results from all three platforms to try to capture subtle but consistent signals. We applied the method of Rank Products (RP) [41] to lists of genes ranked by either fold change or SAGAT. Table 3 shows results from this procedure. As we wanted to evaluate only the most confident predictions, we corrected for multiple testing by controlling the PFER (per family error rate). This is a strict multiple hypothesis test correction method that is generally more conservative than the FDR (false discovery rate) or FWER (family wise error rate) [42]. A total of 19 genes were found to be significantly DE at a PFER of .05. When ranking genes by fold change before applying RP, 12 genes were found to be significantly DE—five upregulated and seven downregulated. When using SAGAT to rank the genes instead, 18 genes were significantly DE—seven upregulated and 11 downregulated. SAGAT with RP detected all but one of the genes found using fold change with RP, and seven genes were identified only through use of SAGAT. We refer to the 11 genes detected by both fold change and SAGAT rankings as Group I; Group II genes are those that were detected exclusively using SAGAT.

**Table 3:** Insulin resistance significant DE genes.

Symbol	Description	Direction	SAGAT PFER	FC PFER
FOSB*	FBJ murine osteosarcoma viral oncogene homolog B	Up	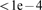	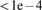
ACTG2	actin, gamma 2, smooth muscle, enteric	Down	0.0007	0.0007
FADS1*	fatty acid desaturase 1	Down	0.0022	0.0048
**PMP2**	**peripheral myelin protein 2**	**Down**	**0.0034**	**0.0770**
**ATP1A2** *	**ATPase, ** 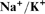 ** transporting, alpha 2**	**Down**	**0.0040**	**0.1620**
CNN1	calponin 1, basic, smooth muscle	Down	0.0067	0.0114
CSN1S1	casein alpha s1	Down	0.0123	0.0004
SELE*	selectin E (endothelial adhesion molecule 1)	Up	0.0174	0.0006
CASQ2	calsequestrin 2 (cardiac muscle)	Down	0.0176	0.0238
FAM150B	family with sequence similarity 150, member B	Down	0.0188	0.0006
**FASN** *	**fatty acid synthase**	**Down**	**0.0231**	**0.0610**
**FOS** *	**v-fos FBJ osteosarcoma viral oncogene homolog**	**Up**	**0.0242**	**0.0885**
**SRGN**	**serglycin**	**Up**	**0.0249**	**0.6952**
CILP	cartilage intermediate layer protein	Up	0.0271	0.0420
**CXCR4** *	**chemokine (C-X-C motif) receptor 4**	**Up**	**0.0311**	**0.6058**
PPBP*	pro-platelet basic protein (chemokine ligand 7)	Down	0.0325	0.0355
AADAC	arylacetamide deacetylase (esterase)	Up	0.0374	0.0019
**ELOVL6** *	**long chain fatty acid elongation**	**Down**	**0.0425**	**0.0734**
*IL6* *	*interleukin 6 (interferon, beta 2)*	*Up*	*0.1340*	*0.0120*

Significance was determined by running the Rank Products (RP) algorithm on ranked lists of genes derived from Affymetrix, Agilent, and Illumina microarray data. Genes were ranked by both fold change and SAGAT before applying RP; the PFER (per family error rate) is displayed for both cases. Only those genes with a PFER of .05 or smaller (achieved using SAGAT or fold change) are considered significant. Genes in normal font were significant using both fold change and SAGAT, genes in bold were identified only with SAGAT, and IL6 was found only with the fold change metric.

*Literature evidence implicates gene with insulin resistance, diabetes, or fatty acid metabolism.

We searched the literature for evidence implicating the genes of Table 3 in insulin resistance, diabetes, or fatty acid metabolism (an important function of adipose tissue). Genes for which evidence was found are marked with an asterisk. Four of the Group I genes [FOSB (Entrez Gene ID: 2354), FADS1 (3992), SELE (6401), PPBP (5473)] had some literature describing their involvement; five of the Group II genes [ATP1A2 (Entrez Gene ID: 477), FASN (2194), FOS (2353), CXCR4 (7852), ELOVL6 (79071)] were also implicated.

**Figure 6:**
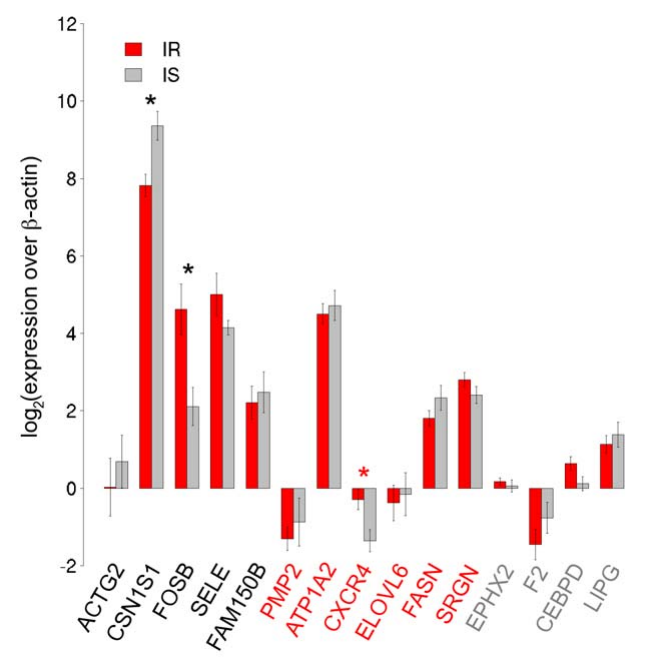
Quantitative PCR validation of insulin resistance candidate genes. Fifteen genes were tested for differential expression between 11 out of the original 12 insulin resistant samples and all 12 original insulin sensitive samples using TaqMan Real-time PCR. The first five genes came from predictions of both fold change and SAGAT, the next six were suggested by SAGAT only, and the last four genes served as negative controls. The directionality of differential expression of all non-control genes was in agreement between the microarray and qPCR data. Three DE genes were statistically significant according to qPCR: CSN1S1, FOSB, and CXCR4.

To experimentally validate these candidates, we performed quantitative RT-PCR (qPCR) using 23 of the original 24 RNA samples subjected to an amplification reaction. We tested 11 of the 19 genes from Table 3: five from Group I and six from Group II. We also tested four genes that were not significant by Rank Products; these genes serve as negative controls. For each gene, we calculated the mean 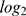 fold change over the 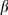-actin (Entrez Gene ID: 60) housekeeping gene for the insulin resistant and insulin sensitive samples. Results are displayed in Figure 6. Of the Group I and II genes tested, all had qPCR expression differences that matched the direction of those identified using Rank Products.

We then tested the significance of each gene's expression difference using a Wilcoxon rank-sum test. Three of the genes had p-values smaller than a .05 threshold: CSN1S1 (Entrez Gene ID: 1446), FOSB, and CXCR4 (marked by asterisks in Figure 6). The first two genes are from Group I; the third is from Group II. Of the four negative controls tested, none were found significantly different in expression between the two groups.

## Discussion

In this work, we present SAGAT, a principled method for integrating pre-existing microarray knowledge with a dataset of interest to identify DE genes. From prior knowledge, SAGAT extracts “eigengenes”, or mathematically independent transcriptional modules, which collectively describe observed expression dependencies between genes. These dependencies are combined with the expression changes of each gene in the data to form the SAGAT score, which enables expression information to be shared between genes that are coexpressed in the knowledge.

To validate SAGAT, we first demonstrated that a compendium of microarray knowledge showed significant modularity. This result, which was not sensitive to varying compendium sizes (not shown), was not surprising, as it has been shown before on knowledge sets of a smaller scale. Nevertheless, it was not clear whether such modules would be detectable on a much larger and more heterogeneous collection of microarrays.

Next, we demonstrated favorable SAGAT performance in identifying DE genes on a series of simulated datasets. We note that our model for simulating data represents an oversimplification of realistic coexpression relationships between genes (see Materials and Methods), but with it we can create distinct numbers of modules in DE and non-DE genes to test the limits of SAGAT performance. As detailed in the Results, SAGAT most improves performance with respect to the fold change metric when transcriptional modules are only composed of DE genes. As the number of non-DE gene modules increases, the performance improvement decreases, but at a realistic ratio of DE gene modules to non-DE gene modules (Figure 2B, which closely matches the configuration of the prostate cancer dataset), SAGAT still outperforms fold change for all numbers of replicates tested.

We evaluated SAGAT on three highly replicated microarray datasets. We chose datasets with many replicates so we could approximate a gold standard DE gene list for each one. Ideally, results from an independent and more accurate experiment like quantitative RT-PCR would provide the DE gene truth for a given dataset, but quantifying expression differences of every gene on a microarray would be prohibitively expensive. Instead, we assume that for each of the three datasets, the number of replicates is large enough that DE genes calculated using fold change on all arrays is approximately correct. Then the task becomes using small (often noisy) subsets of each dataset to predict the true DE genes. We applied the fold change, *limma* t, and SAGAT metrics to multiple non-overlapping subsets of varying numbers of replicates. SAGAT always outperforms the t-statistic, often by a large margin. With sample sizes of only 1 replicate, the *limma* t is not applicable as it requires a fold change variance estimate. Compared to fold change, SAGAT nearly always better identifies DE genes; in the worst case it leaves performance unchanged. These results suggest that SAGAT would be consistently beneficial for predicting DE genes from a dataset of interest. Importantly, the results displayed in Figure S4 demonstrate that use of SAGAT leads to improved statistical power at a small fixed false positive rate, which is a necessity for the effective analysis of high-throughput biological experiments.

We expressed SAGAT's performance improvement over fold change in terms of the effective number of arrays added. This shows that, except in a small number of cases, use of SAGAT always increases the effective sample size of an experiment. In some cases this increase is substantial: for one two-array subset of the prostate cancer dataset, the effective sample size became 4.72 arrays, or more than double the initial sample size of the experiment. As expected, the number of arrays added decreases as the initial number of arrays increases, due in part to the lower capacity for prediction improvement when starting with a larger sample size.

We also demonstrated that SAGAT outperforms the related GEO method when evaluated on the prostate cancer dataset. As even the fold change method consistently outperforms the GEO method, it appears that more accurate estimation of gene variances is not the most effective way to improve performance for this dataset. In contrast, use of gene module information from an SVD of microarray knowledge gives consistent improvement over fold change.

We determined the sensitivity of SAGAT performance to the number of arrays in the knowledge compendium. It was shown in [11] that the GEO method does not give further performance improvement when knowledge exceeds 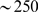 arrays. To compare, we evaluated the effect of compendium size on SAGAT performance using the prostate cancer dataset. Unlike the GEO method, SAGAT continues to improve performance as the compendium increases in size. The improvement starts leveling off near the compendium's full size (4400 arrays), but an even larger knowledge compendium should still give better performance. Thus, SAGAT is able to extract useful information from much larger microarray compendia than the GEO method.

Given SAGAT's potential to improve DE gene identification, we applied the method to a novel insulin resistance dataset obtained from three different microarray platforms. An initial attempt to identify DE genes on each platform separately yielded no candidates, suggesting that the transcriptional response in question was noisy and/or subtle. A Gene Ontology term enrichment analysis on data from each platform consistently identified terms related to immune response (results not shown), implying that a reproducible biological signal was present in the data. To improve the signal to noise ratio at the gene level, we used the method of Rank Products (RP) across all three platforms to identify subtly but consistently changing DE genes.

An application of RP to genes ranked by fold change yielded 12 DE gene candidates with a per family error rate of .05 or smaller. A similar analysis on genes ranked by SAGAT yielded 18 genes, 11 of which overlapped with the fold change list. This suggests that the incorporation of transcriptional module information resulted in an increased sensitivity to detecting DE genes. We intentionally used a very strict significance threshold to select a small number of DE genes that were most consistently changed (and which hopefully represent true biological differences), but relaxation of this threshold would lead to additional candidates.

We next performed a literature search on each significant gene for information implicating it in insulin resistance, diabetes, or fatty acid metabolism. This uncovered evidence for multiple genes from three biological processes: inflammation [SELE, IL6 (Entrez Gene ID: 3569), PPBP, CXCR4], cell differentiation [FOSB, FOS], and fatty acid synthesis [FADS1, FASN, ELOVL6] [43]–[45]. A role for inflammation in IR has previously been suggested by a similar study [35], but of the four pro-inflammatory genes listed above only IL6 was also detected in that work. In this study, SELE, IL6, and CXCR4 were upregulated in insulin resistant patients, reinforcing the positive role of inflammation in IR.

Cell differentiation has also been implicated in insulin resistance in the sense that insulin resistant adipose tissue displayed lower expression of differentiation markers than their insulin sensitive counterparts [37]. In this work FOSB and FOS were upregulated in IR, which is compatible with the above since both gene products have been shown to trigger de-differentiation [46],[47].

Fatty acid synthesis has long been known to be relevant to insulin resistance [48]. The details of this relationship are not always consistent: FADS1 is known to be downregulated in IR [49], while ELOVL6 has shown the opposite effect [50] and FASN has shown conflicting results [51]. To our knowledge, no single study has analyzed the effects of all three of these fatty acid synthesis genes with respect to insulin resistance in adipose tissue. Our results show a coherent decrease in the gene expression of all three genes, suggesting that obesity-independent insulin resistance is associated with altered fatty acid synthesis and storage in adipose tissue. We speculate that such an occurrence may lead to inappropriate fatty acid accumulation elsewhere (i.e. circulating in serum), which has been known to lead to IR [51]. One explanation for the inconsistent results in previous studies is the potentially confounding effects of obesity (a condition where fatty acid synthesis increases) and insulin resistance. The current study explicitly attempts to remove the former effect.

Taken together, the above results emphasize the importance of increased inflammation, differentiation, and decreased fatty acid synthesis to adipose tissue-based insulin resistance. We note that our confidence in this assertion was greatly helped by SAGAT, as four of the nine genes involved in these processes were only identified using this method. This is particularly true for genes like CXCR4, whose PFER received a substantial boost upon application of SAGAT (0.6058 to 0.0311). We expect that further experimentation will reveal the precise relationships between these processes and IR.

The remaining significant genes detected only by SAGAT exhibited varying levels of insulin resistance-related literature evidence. ATP1A2, which codes for an ATPase, was previously found to be differentially expressed between insulin resistant and insulin sensitive muscle tissue, though in the opposite direction than was found in this study [52]. PMP2 (Entrez Gene ID: 5375) and SRGN (5552), coding for a myelin protein and hematopoietic proteoglycan, respectively, lack any literature evidence for a relationship to IR; illumination of their specific roles would require further study.

To confirm the validity of some of the above DE gene candidates, we performed qPCR using RNA samples from 23 of the original 24 patients (one IR sample did not have sufficient RNA for the procedure). We tested five genes found to be significant using both fold change and SAGAT, six genes found only with SAGAT, and four negative controls. All of the qPCR expression differences of the non-control genes matched the direction of those from the microarray data, suggesting that these changes are reproducible. We then tested the significance of these changes using a Wilcoxon rank-sum test (RST). We note that the RST is one of the more conservative two-sample tests available [53], and we anticipated noisy data due to the amplification reactions needed prior to qPCR (see Materials and Methods section). Nevertheless, three genes—two identified by fold change and SAGAT, one by only SAGAT—were found to be significant. In contrast, none of the negative control genes showed significant expression differences. Combining the qPCR results together with the literature evidence implicating four of the eight genes not confirmed by qPCR suggests a false positive rate of 0.4 (2/5) for fold change and 0.36 (4/11) for SAGAT. Though the difference between these values may not be statistically significant, this result suggests that SAGAT was able to improve the sensitivity of DE gene detection in this experiment without increasing the false positive rate. We did not explicitly test IL6 using qPCR, although we note that previous work has shown this gene to be overexpressed in insulin resistant adipose tissue [35]. This is the only gene detected using fold change that was not also detected using SAGAT, which may reflect discordant expression patterns of IL-6 between previously existing datasets and this one.

We now explore the means by which SAGAT improves prediction of DE genes. Results from the simulation study demonstrate that the method improves performance to the extent that DE genes are more likely to be in transcriptional modules than non-DE genes. This is realized through the standard error term (denominator) of the SAGAT score (see Materials and Methods). For a given gene in a module (eigengene), the standard error for that gene's mean expression difference receives contributions from measurements of the other genes in that module, leading to a smaller error (more precise estimate of expression). Thus, genes in modules will on average have slightly boosted SAGAT scores compared to genes acting in isolation. In the process of characterizing modularity of the HGU95Av2 knowledge set to parameterize our simulation, we have discovered that DE genes are more likely to be in modules than non-DE genes. Given that the performance improvements in the letrozole treatment and colorectal cancer datasets were similar to the prostate cancer case, we expect this feature of DE genes (and the corresponding performance improvement by SAGAT) to be generalizable to a wide variety of biological datasets. To support this hypothesis, we note that genes which are frequently differentially expressed are more likely to be associated with a disease [54], and genes implicated in the same disease show higher levels of coexpression (modularity) than randomly selected genes [55].

A closer look at the functional form of the SAGAT score shows its similarity to versions of the t-statistic, including the *limma* t and SAM [39],[56]. The difference between these metrics lies in their method for calculating the standard error of each gene's mean expression difference. Though the *limma* t-statistic borrows information for calculating this term from other genes, SAGAT is the only approach that identifies and uses expression dependencies between genes in the computation of gene-wise variances. Fortunately, this addition is not computationally expensive, as SAGAT utilizes efficient algorithms. Eigengenes are identified using SVD, which must only be run once per knowledge compendium. Computation of the SAGAT score requires projection of a small (with respect to the size of the knowledge) dataset into eigengene space followed by a simple dot product for each gene. Practically, the running time of SAGAT is approximately the same as that of related methods like the *limma* t-statistic. We note, however, that the distribution of the SAGAT score is complex, and unlike the t-statistic, it does not provide for a straightforward estimation of statistical significance. Thus, we advocate data permutation-based methods (similar to those used by SAM) to calculate SAGAT p-values.

Use of SAGAT does require some explicit assumptions about microarray knowledge. First, we assume that (detectable) multi-gene transcriptional modules give rise to the expression values in a compendium of microarray knowledge. Previous work [12]–[18] detecting reproducible, biologically plausible transcriptional modules (along with results from our characterization of the HGU95Av2 compendium) suggest that this is a valid assumption. Second, representing the transcriptional levels of each gene as a weighted combination of eigengene levels assumes that each gene's expression can be modeled in a linear fashion. While some evidence exists to support this assumption [57], it is more realistic that expression is a non-linear phenomenon. Nevertheless, linear approximations have proven useful and even quite accurate in the modeling of non-linearity [58]. We find empirical support for this accuracy in the coherence of the GO terms significantly enriched in eigengenes of the HGU95Av2 compendium. Third, though SVD does not make any distributional assumptions about the knowledge, the analytical derivation of the SAGAT score requires the eigengene expressions to be statistically independent. When the underlying eigengenes are distributed as multivariate normal (MVN) random variables, they will exhibit independence, but otherwise this may not be the case. Given that we did not explicitly enforce this assumption in either the simulated data (here, genes were MVN, not eigengenes) or the highly replicated real datasets, this assumption does not appear to be detrimental to SAGAT performance.

An implicit assumption in the use of prior microarray knowledge to inform a novel dataset is that the expression dependencies from the knowledge are conserved in the novel dataset. In a worst-case scenario, a novel dataset would exhibit a transcriptional response completely unlike anything assayed previously. Given the modular nature of transcription, we expect this to be unlikely, and the favorable performance of SAGAT on three independent biological datasets supports this assertion. Additionally, as even more microarray experiments are performed and their data become available, the likelihood of such a scenario occurring will tend to zero.

As SAGAT requires a large compendium of microarray knowledge, it is worth examining potential biases in currently available compendia. Due to their popularity among researchers, the vast majority of publicly available human microarray datasets are from Affymetrix platforms. Thus, the three compendia and highly replicated datasets used in this study represent the three most popular human Affymetrix GeneChips. One concern would be that a non-biological bias (perhaps due to cross-hybridization between specific probesets) exists in Affymetrix data which cannot be detected and removed without considering data derived from other platforms. This might lead to artifactual coexpression relationships. Another concern would be that the dependency information inferred from Affymetrix microarray knowledge is not extensible to non-Affymetrix datasets, due to differences in probesets or the artifactual coexpression phenomenon discussed above. While these concerns may have some merit, we note that in applying SAGAT to a novel insulin resistance dataset we incorporated microarray knowledge from an Affymetrix platform with data from Affymetrix, Illumina, and Agilent platforms. Given the ability of SAGAT to correctly identify novel DE genes in this case, we do not believe such a large Affymetrix-specific bias is present.

Finally, as the value for parameter *M* (specifically, the fraction *M*/*P*—see Materials and Methods) was set for all three Affymetrix compendia based on performance observed using the HGU95Av2 compendium, there is an implicit assumption that the optimal parameter value is identical between platforms. We evaluated this by testing SAGAT on several data subsets from the HGU133A and HGU133plus2.0 platforms across a range of *M* values. Results suggest that a value of *M* that is approximately half the number of arrays in the compendium is nearly optimal for all three compendia (not shown). Nevertheless, more principled approaches of effectively choosing platform-specific values for *M* likely exist, and future work will include identifying these approaches.

We provide SAGAT as an R package (*sagat*), which is available at https://simtk.org/home/sagat. The package includes all necessary functions to run the method along with preprocessed versions of the **W** matrix for the three Affymetrix platforms analyzed in this work. Given its abilities to improve the prediction of DE genes, we expect that SAGAT will be useful to microarray researchers studying a wide range of biological phenomena.

## Materials and Methods

### Ethics Statement

The insulin resistance study was approved by the Stanford University Human Subjects Committee and the National Institute of Digestive Diseases and Kidney Disease (NIDDK) Institutional Review Board, and all subjects gave written informed consent.

### Modularity of Gene Expression Data

We downloaded all available expression data for the Affymetrix HGU95Av2 microarray (GPL91) from the Gene Expression Omnibus (GEO: http://www.ncbi.nlm.nih.gov/geo/) in August 2007. These data are hereafter referred to as “knowledge”, or a knowledge compendium. The Robust Multi-array Average (RMA) algorithm was first used to compute averages between probes in a probeset. Probesets were then mapped to a non-redundant list of Entrez Gene IDs (provided by the Bioconductor R package *hgu95av2* version 1.16.0), and expression values for multiple probesets of the same gene were averaged using an arithmetic mean. This resulted in a matrix of 9105 genes by 4440 arrays, which is available for download at https://simtk.org/home/sagat. We log transformed and quantile normalized the arrays to ensure that they were on the same scale, and we computed the gene-gene covariance matrix across all 4440 arrays, ignoring missing values. In order to simplify characterization of the covariance structure, we discretized the covariance matrix such that diagonal entries and entries whose absolute value was greater than the mean covariance value (.25) were set to one, and all others were set to zero. We then hierarchically biclustered the rows and columns of the binarized covariance matrix (using a distance metric of 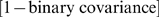 and complete linkage) to enable visualization of gene groups with significant covariances. Here, we define an expression module as a group of genes of size 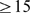, identified upon hierarchical biclustering of the covariance matrix, whose pairwise binarized covariance values are all nonzero.

To test whether the observed modularity was due to chance, we generated 100 permuted versions of the knowledge matrix, whereby the columns of each row were permuted independently of the other rows. We followed the subsequent steps of calculating covariance, discretizing, and clustering as above, and we counted the number of diagonal covariance clusters containing 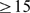 genes (i.e. expression modules).

To characterize expression modularity with respect to differentially expressed (DE) or non-DE genes, we coupled the HGU95Av2 compendium with a human prostate cancer microarray dataset [38]. Beginning with the clustered, binarized covariance matrix of Figure 1B, we generated five 1000-gene covariance matrices by randomly subsetting the full matrix. In each one, we zeroed all covariance values in off-diagonal clusters and those in diagonal clusters with fewer than five genes (in the 1000-gene matrix, we relax the cutoff for expression modules to five genes). We calculated the mean number of genes per module in the remaining covariance modules across the five matrices and used this for simulating new compendia (details below). Using the prostate cancer dataset, we identified DE genes as those having a *limma* t-statistic with FDR 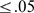 (calculated with the *limma* R package version 2.8.1). We split each of the five covariance matrices above into DE or non-DE subsets, and we calculated the mean percentages of genes in covariance modules for each. These values were also used for simulating compendia (below).

### Singular Value Decomposition (SVD) of Gene Expression Data

**Figure 7:**
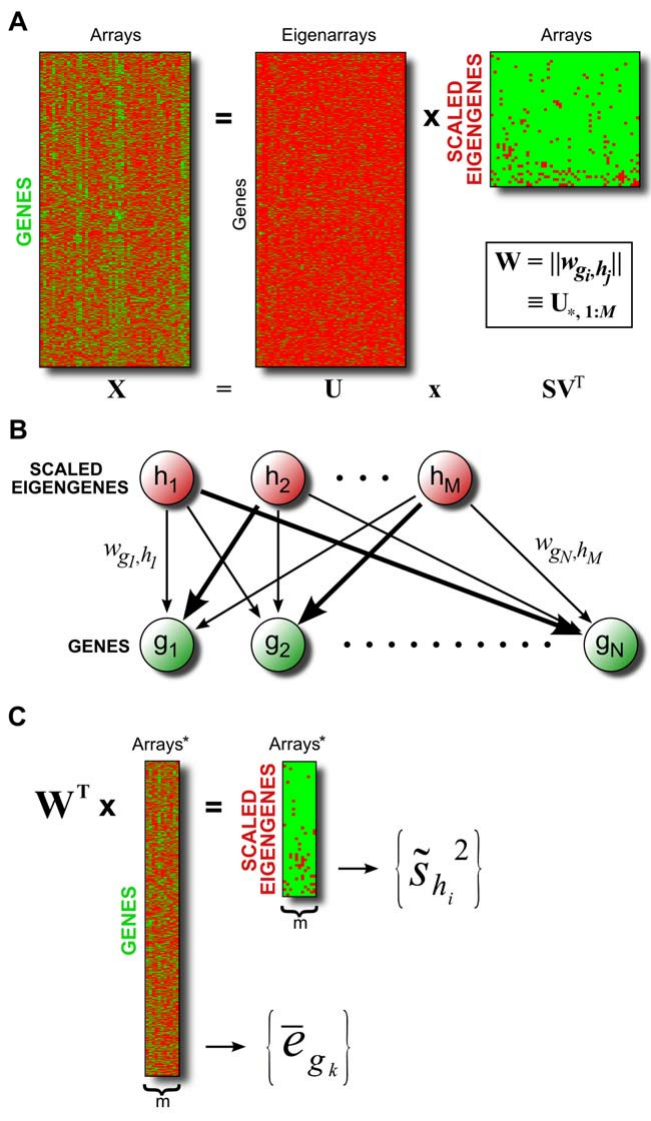
Overview of SAGAT method. (A) Schematic of SVD applied to a matrix of microarray knowledge. The dimensions of matrix 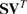 are “scaled eigengenes” by arrays. The weight matrix **W**, consisting of the first *M* columns of **U**, is used in subsequent steps. (B) Graphical depiction of SVD model, whereby each gene's expression is a weighted combination of underlying eigengene expressions with weights given by **W**. (C) SAGAT transforms a dataset of interest into eigengene space by premultiplying the data matrix by the transpose of the weight matrix **W**. Both the original and transformed data matrices are subsequently used to calculate the SAGAT statistic (in the forms of 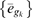 and 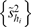, respectively).

An overview of the SVD procedure is illustrated in Figure 7A. In equation form, SVD transforms an 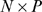 (genes×arrays) knowledge matrix **X** into the product of three matrices **U**, **S**, and **V**:
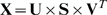
(1)where 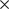 and *^T^* represent matrix multiplication and transposition, respectively. As detailed in [22], the dimensions of **U**, **S**, and 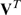 are genes×eigenarrays, eigenarrays×eigengenes, and eigengenes×arrays, respectively. We follow the notation used in [59] and treat the dimensions of the product 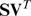 as “scaled eigengenes”×arrays. As SVD requires complete data, we either exclude arrays of the knowledge matrix with missing values (if fewer than 10% of the total number of arrays are incomplete) or impute missing values using the K-nearest neighbor algorithm implemented in the *impute* R package (version 1.6.0) [24]. We center and scale the rows of the complete data matrix and run the *svd* R function.

To confirm the validity of an eigengene theory of gene expression, we first ran SVD on the HGU95Av2 knowledge matrix with missing values imputed. We then identified enriched Biological Process Gene Ontology terms for each eigengene by applying the Kolmogorov-Smirnov statistic (implemented in the *topGO* R package version 1.2.1). Specifically, within each eigengene, all 9105 genes were ranked (in descending order) by the magnitudes of their weights (determined from the appropriate column of **U**). GO terms significantly enriched at the top of each ordered list were then identified using the *getSigGroups* R function.

### SAGAT—*S*VD *A*ugmented *G*ene Expression *A*nalysis *T*ool

SVD constructs a linear relationship between genes and eigengenes such that each gene's expression can be formulated as a linear combination of the eigengene expressions (Figure 7B). We can explicitly represent this in equation form by approximating (1) as follows:
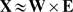
(2)where **W** is simply a matrix containing the first *M* columns of **U** (*M* most significant eigenarrays), and **E** is the product of the first *M* rows of matrix **S** with 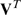. Intuitively, **E** represents the knowledge matrix **X** transformed from array space into eigenarray space, and **W** provides the map between genes and scaled eigengenes. Given a novel dataset **D** with *m* replicates (referred to as “data”), we obtain data-specific eigengene expressions by solving the following approximation for 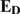:
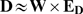
(3)where we use **W** from (2), and 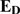 represents dataset **D** transformed into eigengene space. We obtain a mathematically rigorous solution to (3) by premultiplying both sides by the transpose of **W**. This is possible due to the orthogonality properties of SVD and is equivalent to a projection using the pseudoinverse of **W**. Such a projection gives the optimal (in the least squares sense) approximation of dataset **D** in terms of the knowledge set **X**. We note that pseudoinverse projection has previously been successfully used in other areas of microarray analysis, particularly with respect to noise reduction in data [29],[30],[60]. Knowledge of 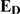 (and **D**) allows us to calculate a mean log expression ratio for each gene 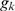 (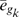) and a log expression ratio sample variance for each eigengene (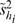) (Figure 7C).

To perform hypothesis tests for differential expression, we created a probabilistic model for each gene's mean log expression ratio 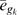. The properties of SVD allow us to approximate this quantity in the following manner:
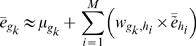
(4)where 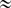 implies “approximately equal to”, 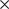 represents scalar multiplication, 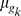 represents the unknown true mean log expression ratio of gene 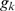, *M* is the number of eigengenes used to reconstitute the gene expressions, the weights 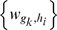 come from **W**, and the 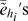 are mean log ratios for mean-centered eigengenes (assumed to be normally distributed):
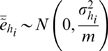
(5)where 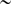 implies “distributed as”, 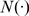 specifies a normally distributed random variable, and 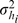 represents the population expression variance for eigengene 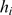. Thus, the 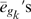 acquire the following distribution:
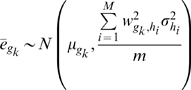
(6)


By using the empirical Bayes variance estimators 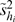 (calculated using the *limma* R package (version 2.8.1) [39]) in place of the unknown 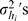, we arrive at the test-statistic 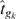 for gene 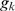, analogous to the one sample t-statistic:
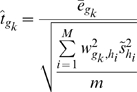
(7)


This “SAGAT score” borrows information regarding expression variability for each gene from covarying genes via their shared eigengenes. Though the statistical model used to derive this metric assumes normally distributed eigengene log expression ratios, it will still provide quantitatively useful scores when this assumption is not met. In the case when 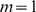, the 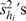 are undefined and a slight modification is required. We discovered that the following form of the SAGAT score gave performance consistent with that achieved on datasets with *m* greater than 1:
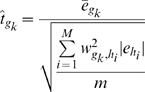
(8)where 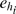 is the single log ratio for eigengene *i* calculated by transforming the data into eigengene space and 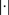 implies absolute value.

In (2), (4), (6), (7), and (8) above, the correct value for *M* is unknown, so we treat it as a parameter to be learned from data. Details of the learning procedure for simulated and highly replicated real data are found below in the corresponding sections.

### Simulation Study

We simulated 1000-gene compendia of microarray knowledge by generating 1000 multivariate normal random variables (using the mvrnorm function in the R *MASS* package version 7.2–48). The mean vector used for the simulation was derived from sample means of 1000 random genes from the HGU95Av2 compendium; the covariance matrix contained all zeros except in positions needed to create the desired modularity structures (Figure 2). In these positions, we used a covariance value of 4, which was chosen to be large enough to generate knowledge compendia that led to noticeable differences in SAGAT performance. We simulated 1000-gene microarray data with numbers of replicates ranging from 1–15 using the procedure listed in [19], parameterized with values derived from the prostate cancer dataset. Each dataset was engineered to contain 100 DE genes.

We ran SVD on each simulated compendium and used the resultant **W** matrix to test SAGAT on all combinations of data and knowledge. To estimate *M*, we evaluated SAGAT performance as a function of varying *M* across a range of simulated data (1–15 replicates) and knowledge compendia (all configurations between Figures 2A and B). We chose a value of *M* that gave optimal performance across all tested configurations; this value was used for all subsequent tests on simulated data. We compared the results of these tests (in the form of ROC AUC and TPR at a fixed FPR of .05) to that achieved by fold change to determine the range of data/knowledge configurations in which SAGAT outperformed fold change.

### Highly Replicated Real Datasets

We evaluated SAGAT's potential to improve DE gene prediction on real data by testing the method on three highly replicated datasets. This approach is similar to that used by [11], except that we choose area under the ROC curve and true positive rate as our evaluation metrics. The first dataset, listed above, measures differences in expression between prostate cancer tissue and matched non-cancer prostate [38]. This dataset measures expression of 9105 genes (identified by mapping probe names to Entrez Gene IDs as above) across 47 pairs of samples (“replicates”: as Affymetrix arrays measure one RNA sample at a time, one experimental replicate is equivalent to two arrays). The second dataset compares breast cancer tissue before and after letrozole treatment [61]. These data were collected across 58 pairs of samples on the HGU133A Affymetrix platform, which measures expression of 13410 Entrez Genes. The final dataset measures expression differences between colorectal cancer tissue and matched non-cancer tissue [62]. This dataset was generated for 32 pairs of samples on the HGU133plus2.0 Affymetrix platform, which encompasses 20099 Entrez Genes. For each dataset we determined truly DE genes by calculating either mean fold changes or *limma* t statistics across all replicates and counting genes with the largest scores (irrespective of sign) as DE. The number of DE genes in each case was set to the number of genes whose t-statistic was significant at a .05 FDR cutoff. We performed all analyses using the *limma* R package (version 2.8.1).

To obtain knowledge for each dataset, we downloaded all publicly available microarray datasets from GEO (minus the highly replicated datasets listed above) for each of the corresponding Affymetrix platforms. As mentioned above, the HGU95Av2 compendium contained 4440 arrays, while the HGU133A (GPL96) and HGU133plus2.0 (GPL570) compendia consisted of 14476 and 12217 arrays, respectively (as of March 2008). For each knowledge source, we either imputed missing data (HGU95Av2) or excluded incomplete arrays (HGU133A, HGU133plus2.0) to arrive at the number of arrays listed above. As with the above datasets, we mapped probe names of each knowledge compendium to the corresponding Entrez Genes. We ran SVD as detailed above on each knowledge matrix, generating the matrices 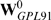, 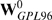, and 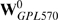, each containing the maximal number of eigengenes.

We evaluated SAGAT on its ability to identify DE genes from subsets of each dataset that best match the truly DE genes discovered using all replicates. For each dataset, we generated the maximal number of non-overlapping subsets of size 1, 2, 5, and 15 (14 for Letrozole treatment) replicates. We ran SAGAT on each data subset with the appropriate **W** matrix (defined below), calculated fold changes and *limma* t-statistics for comparison, and computed the ROC AUCs and TPRs evaluated at FPR = .05 for all three metrics with respect to the truly DE genes. We used the R package *ROCR* (version 1.0–2) [63] for AUC and TPR calculations.

To determine the optimal number of eigengenes (*M* parameter) to use in the **W** matrices for each dataset, we tested all possible numbers of eigengenes from 5 to 4400 (in multiples of 5) on several subsets of the Prostate cancer dataset. The number of eigengenes that gave the best performance overall was used as the value for 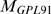, and the values for 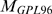 and 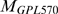 were set such that they yielded an identical fraction of *M/P*, where *P* is the total number of arrays. From these values of *M* we subset the matrices 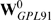, 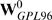, and 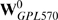 by only including the first *M* columns of each to form 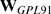, 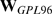, and 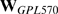, respectively. We used these modified matrices in the SAGAT analysis described above.

We also characterized SAGAT performance in terms of the effective number of arrays added. For each of the highly replicated datasets, we calculated ROC AUCs of the fold change metric applied to all non-overlapping replicate subsets ranging in size from 1 to the total number of replicates. These AUCs enabled us to fit a “standard curve” for each dataset, from which we could interpolate the mean number of arrays gained by using SAGAT given initial numbers of 2, 4, 10, and 30 (28 for Letrozole dataset) arrays [equivalent to 1, 2, 5, and 15 (14) replicates, respectively].

### Comparison to related method

We compared SAGAT performance to that of the GEO method, which was implemented as described in [11] using both the standard method and “voting” scheme. The comparison was made as above on subsets of the prostate cancer dataset, using the HGU95Av2 compendium as knowledge. We also evaluated the effect of smaller compendium sizes on SAGAT performance by taking random subsets of 100 to 4000 arrays (10 subsets per size) of the HGU95Av2 compendium and calculating the mean AUC improvement over fold change across all subsets of the prostate cancer dataset.

### Insulin Resistance Dataset

We applied SAGAT to an unpublished biological dataset investigating human insulin resistance. Briefly, 33 moderately obese but otherwise healthy female patients were tested for insulin resistance using a modified insulin suppression test [64]. RNA was isolated from the adipose tissue of the 12 most and 12 least insulin resistant patients and hybridized to three different microarray platforms: Affymetrix HGU133plus2.0, Agilent G4112A, and Illumina HumanRef-8 v2. The data from the Affymetrix platform were normalized using a bias correction algorithm [65]; data from the other two platforms were normalized using default algorithms accompanying the respective feature extraction programs. Raw data for each of the three platforms are available for download as Datasets S1,S2,S3.

We first used the *limma* t-statistic to identify DE genes using the data from each platform individually. To utilize data from all three platforms simultaneously, we applied the method of Rank Products to lists of genes from each platform ranked either by fold change or SAGAT score (in both cases separating up and downregulated genes).

Predicted DE genes were validated by quantitative RT-PCR experiments. 200ng of total adipose tissue RNA was amplified using the Ambion MessageAmp II aRNA Amplification Kit (cat #AM1751) according to manufacturer's instructions. 1ug of amplified product was then used for quantitative PCR analysis using Taqman primer/probe sets for ACTG2 (Entrez Gene ID: 72), CSN1S1, FOSB, SELE, FAM150B (285016), PMP2, ATP1A2, CXCR4, ELOVL6, FASN, SRGN, EPHX2 (2053), F2 (2147), CEBPD (1052), and LIPG (9388) as well as Human 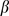-actin endogenous control. Primer/probe sets were purchased from Applied Biosystems (Foster City, CA). Amplification was carried out in triplicate on an ABI Prism 7900HT at 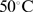 for 2 min and 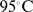 for 10 min followed by 40 cycles of 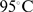 for 15 s and 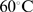 for 1 min. A threshold cycle (CT value) was obtained from each amplification curve and a 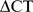 value was first calculated by subtracting the CT value for 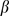-actin from the CT value for each sample. A 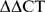 value was then calculated by subtracting the 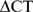 value of a single insulin-sensitive subject (control). Fold-changes compared with the control were then determined by raising 2 to the 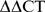 power.

We tested the significance of each gene's qPCR-derived expression differences using a one-sided Wilcoxon rank-sum test (two-sided test was used for negative controls). Genes with p-values smaller than a .05 threshold were considered significant.

## Supporting Information

Figure S1 Comparison of SAGAT and the GEO method. Each panel displays performance on the prostate cancer dataset. (A) The fold change metric was used to rank genes for the gold standard. SAGAT and the GEO method (both with HGU95Av2 compendium) and fold change were run on all combinations of 1, 2, 5, and 15 replicate subsets and the performance improvement of SAGAT and GEO over fold change displayed. (B) Identical conditions as (A), with the limma t metric used to rank gold standard genes. (C) and (D) Similar plots comparing SAGAT and the GEO voting method, which requires two or more replicates to score genes. Fold change and limma t metrics were used to rank gold standard genes in (C) and (D), respectively. In all cases, SAGAT performs as well or better than the GEO method.
(1.72 MB TIF)

Figure S2 Effect of compendium size on SAGAT performance. SAGAT was run on all subsets of the prostate cancer dataset using randomly subset versions of the HGU95Av2 compendium ranging in size from 100 to 4400 arrays. The mean AUC improvement over the fold change method is displayed on the y-axis. Each boxplot shows the results of using 10 random compendium subsets of a given size. Though the rate of performance improvement lessens as the number of arrays in the compendium increases, extrapolation suggests that the addition of more arrays will lead to further improvement.
(7.56 MB TIF)

Figure S3 SAGAT true positive rate for four simulated data-knowledge configurations. In each panel, both SAGAT and the fold change metric were applied to 200 simulated datasets consisting of either one [(A) and (B)] or 15 replicates [(C) and (D)]; the true positive rate (TPR) improvement (evaluated at a fixed false positive rate of .05) achieved by SAGAT over fold change is displayed for each. In (A) and (C), a simulated knowledge compendium matching Figure 2A was used by SAGAT; in (B) and (D) the simulated knowledge corresponds to Figure 2B. SAGAT performance improvements measured by TPR closely resemble those measured by AUC (displayed in Figure 3).
(1.09 MB TIF)

Figure S4 SAGAT, fold change, and limma t TPR performance on subsets of three highly replicated human datasets. In each panel, the gold standard was defined as the top 1122, 588, and 6002 highest scoring genes for the prostate cancer, letrozole treatment, and colorectal cancer datasets, respectively. (A) The fold change metric was used to rank genes for the gold standard. SAGAT (with HGU95Av2 compendium), fold change, and limma t were run on all combinations of n = 1, 2, 5, and 15 replicate subsets and the performance improvement (measured by TPR evaluated at a fixed false positive rate of .05) of SAGAT and limma t over fold change displayed. Limma t requires two or more replicates to score genes. (B) Identical conditions as (A), with the limma t metric used to rank gold standard genes. (C) and (D) Similar plots for the letrozole treatment (HGU133A compendium) and colorectal cancer (HGU133plus2.0 compendium) datasets, respectively. Fold change was used to rank gold standard genes in these panels. SAGAT performance improvements measured by TPR closely resemble those measured by AUC (displayed in Figure 5).
(1.55 MB TIF)

Dataset S1 Affymetrix Insulin Resistance data table.
(6.12 MB TXT)

Dataset S2 Agilent Insulin Resistance data table.
(8.30 MB ZIP)

Dataset S3 Illumina Insulin Resistance data table.
(3.04 MB TXT)
