# Supplementary figures and images for: Using Pre-existing Microarray Datasets to Increase Experimental Power: Application to Insulin Resistance
Source: PLoS Comput Biol. 2010 Mar 26;6(3):e1000718. doi: 10.1371/journal.pcbi.1000718 (PMC2845644; doi:10.1371/journal.pcbi.1000718)

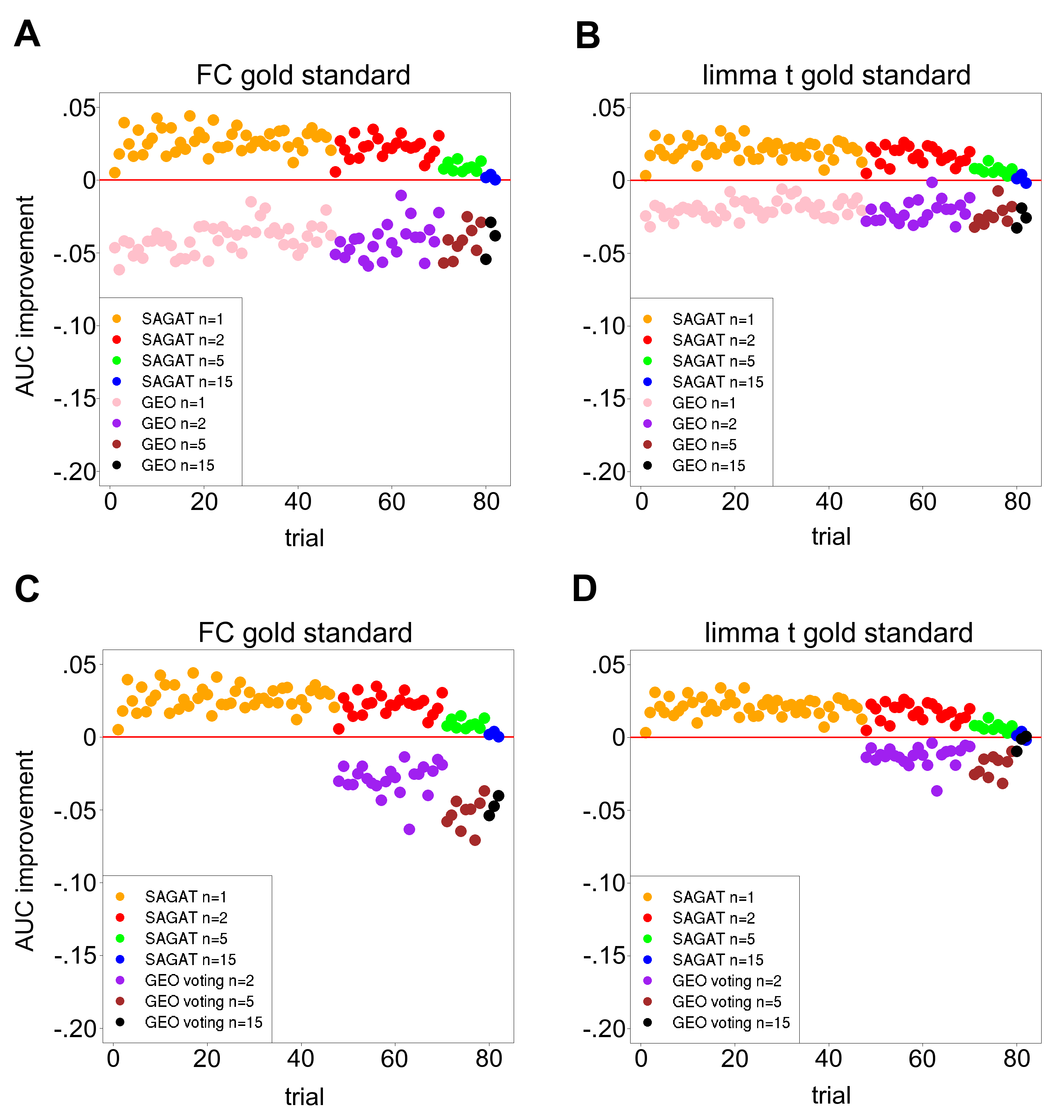

Supplement: Figure S1 — Comparison of SAGAT and the GEO method. Each panel displays performance on the prostate cancer dataset. (A) The fold change metric was used to rank genes for the gold standard. SAGAT and the GEO method (both with HGU95Av2 compendium) and fold change were run on all combinations of 1, 2, 5, and 15 replicate subsets and the performance improvement of SAGAT and GEO over fold change displayed. (B) Identical conditions as (A), with the limma t metric used to rank gold standard genes. (C) and (D) Similar plots comparing SAGAT and the GEO voting method, which requires two or more replicates to score genes. Fold change and limma t metrics were used to rank gold standard genes in (C) and (D), respectively. In all cases, SAGAT performs as well or better than the GEO method. (1.72 MB TIF) [file pcbi.1000718.s001.tif]

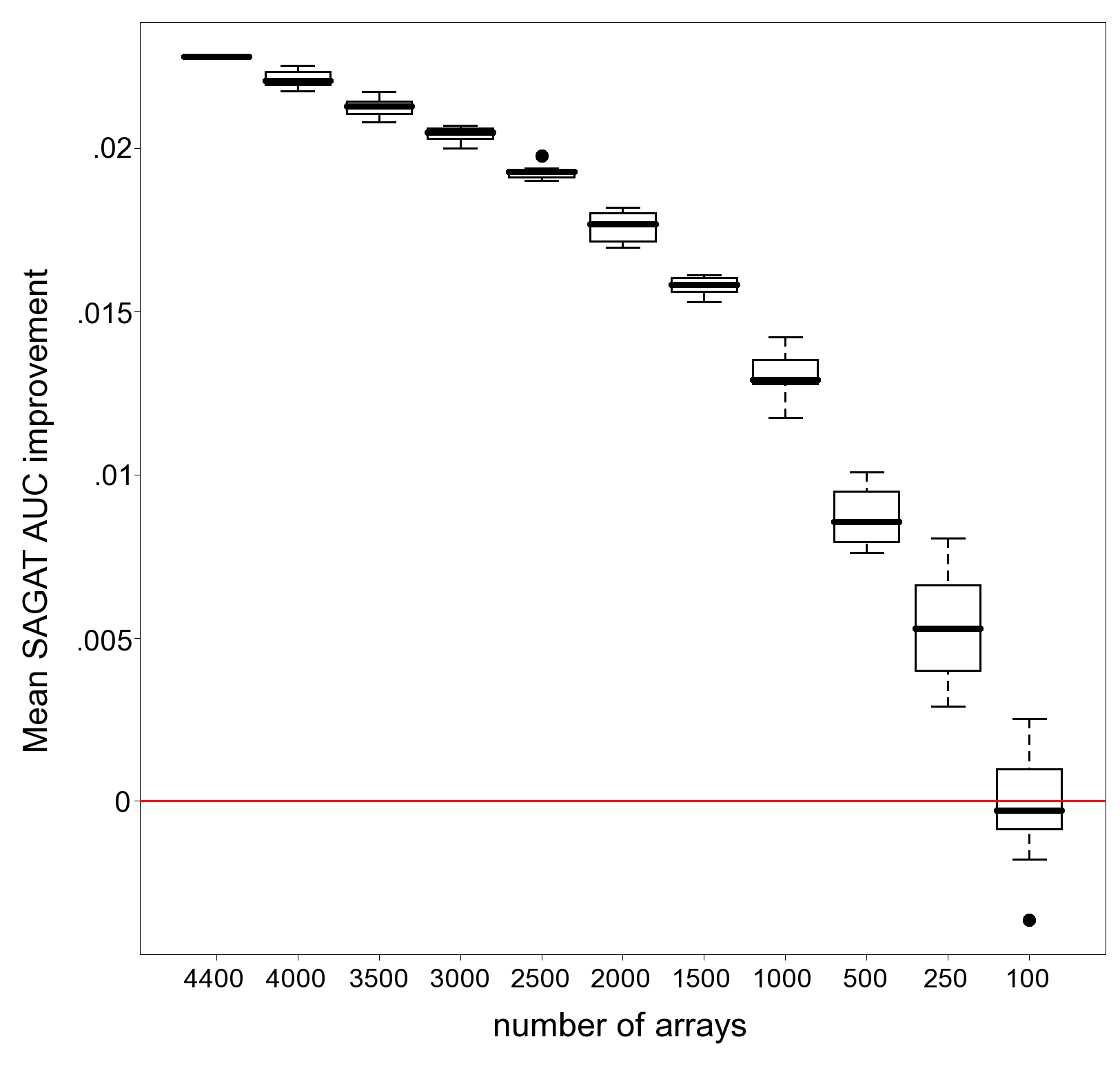

Supplement: Figure S2 — Effect of compendium size on SAGAT performance. SAGAT was run on all subsets of the prostate cancer dataset using randomly subset versions of the HGU95Av2 compendium ranging in size from 100 to 4400 arrays. The mean AUC improvement over the fold change method is displayed on the y-axis. Each boxplot shows the results of using 10 random compendium subsets of a given size. Though the rate of performance improvement lessens as the number of arrays in the compendium increases, extrapolation suggests that the addition of more arrays will lead to further improvement. (7.56 MB TIF) [file pcbi.1000718.s002.tif]

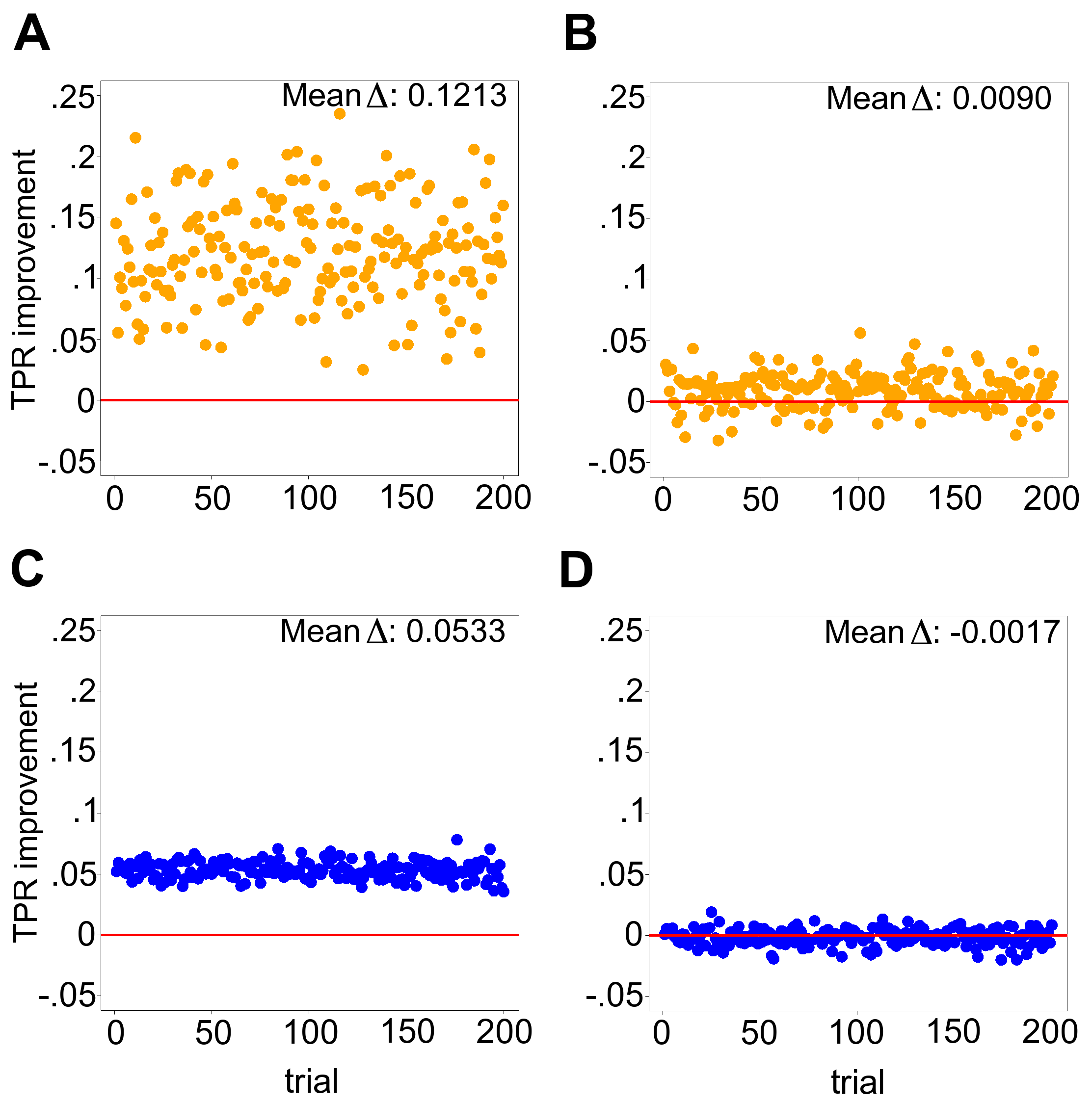

Supplement: Figure S3 — SAGAT true positive rate for four simulated data-knowledge configurations. In each panel, both SAGAT and the fold change metric were applied to 200 simulated datasets consisting of either one [(A) and (B)] or 15 replicates [(C) and (D)]; the true positive rate (TPR) improvement (evaluated at a fixed false positive rate of .05) achieved by SAGAT over fold change is displayed for each. In (A) and (C), a simulated knowledge compendium matching Figure 2A was used by SAGAT; in (B) and (D) the simulated knowledge corresponds to Figure 2B. SAGAT performance improvements measured by TPR closely resemble those measured by AUC (displayed in Figure 3). (1.09 MB TIF) [file pcbi.1000718.s003.tif]

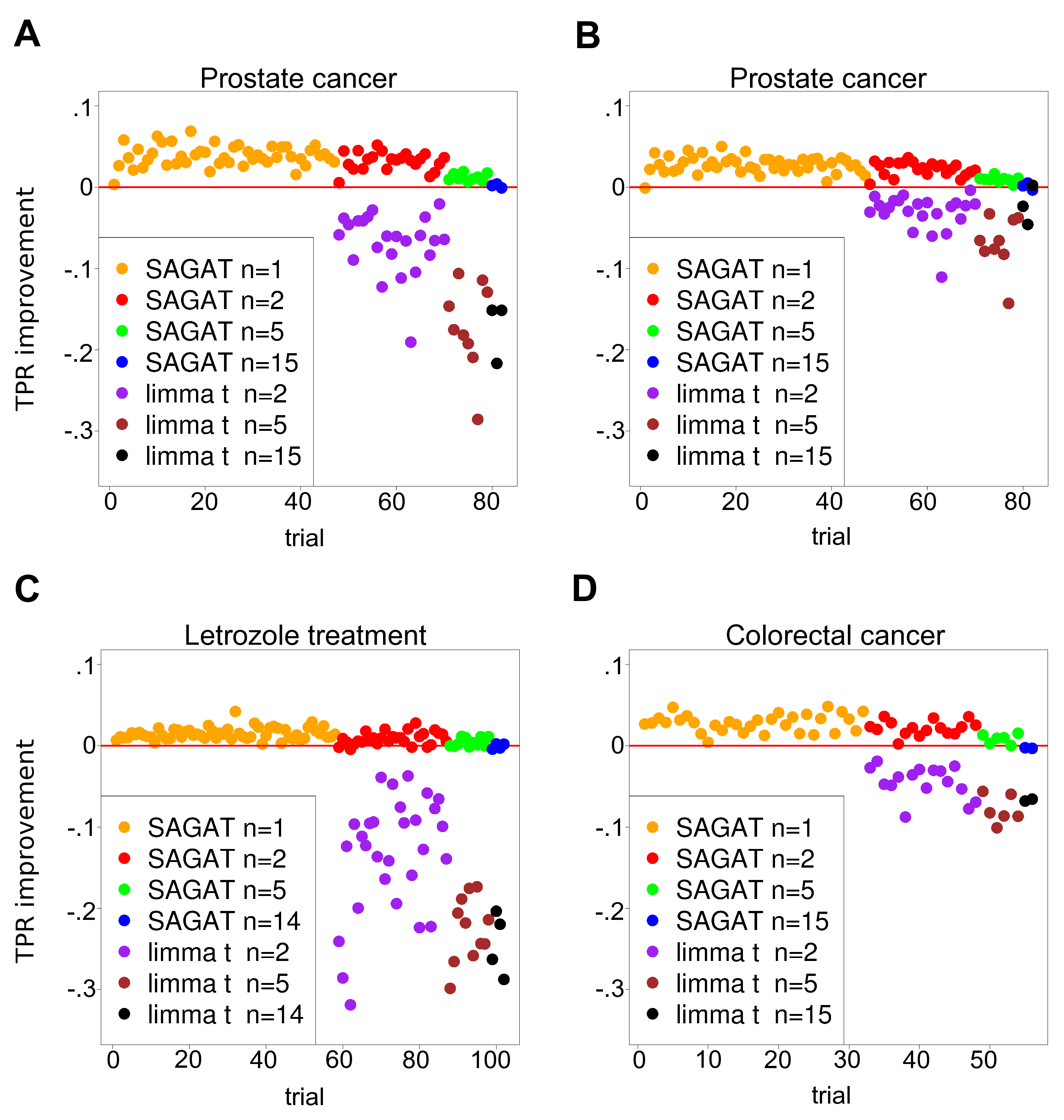

Supplement: Figure S4 — SAGAT, fold change, and limma t TPR performance on subsets of three highly replicated human datasets. In each panel, the gold standard was defined as the top 1122, 588, and 6002 highest scoring genes for the prostate cancer, letrozole treatment, and colorectal cancer datasets, respectively. (A) The fold change metric was used to rank genes for the gold standard. SAGAT (with HGU95Av2 compendium), fold change, and limma t were run on all combinations of n = 1, 2, 5, and 15 replicate subsets and the performance improvement (measured by TPR evaluated at a fixed false positive rate of .05) of SAGAT and limma t over fold change displayed. Limma t requires two or more replicates to score genes. (B) Identical conditions as (A), with the limma t metric used to rank gold standard genes. (C) and (D) Similar plots for the letrozole treatment (HGU133A compendium) and colorectal cancer (HGU133plus2.0 compendium) datasets, respectively. Fold change was used to rank gold standard genes in these panels. SAGAT performance improvements measured by TPR closely resemble those measured by AUC (displayed in Figure 5). (1.55 MB TIF) [file pcbi.1000718.s004.tif]
